# A Sustainable Approach for the Valorization of Underutilized Date Fruits

**DOI:** 10.3390/molecules28155807

**Published:** 2023-08-01

**Authors:** Amel Hamdi, Isabel Viera-Alcaide, Susana Costa, Teresa Lino-Neto, Rafael Guillén-Bejarano, Rocío Rodríguez-Arcos, Ana Jiménez-Araujo

**Affiliations:** 1 Instituto de la Grasa, Consejo Superior de Investigaciones Científicas (CSIC), Campus Universidad Pablo de Olavide (UPO), Building 46, Carretera de Utrera Km1, 41013 Sevilla, Spain; amelhamdi1988@yahoo.fr (A.H.); iviera@ig.csic.es (I.V.-A.); rguillen@ig.csic.es (R.G.-B.); rrodri@ig.csic.es (R.R.-A.); 2 Molecular Biology and Biochemical Engineering Department, Centro Andaluz de Biología del Desarrollo (CABD), University Pablo de Olavide (UPO), CSIC/UPO/Junta de Andalucía, Carretera de Utrera Km 1, 41013 Sevilla, Spain; 3 Centre of Molecular and Environmental Biology (CBMA), University of Minho, Campus de Gualtar, 4710-057 Braga, Portugal; su.costa1993@gmail.com (S.C.); tlneto@bio.uminho.pt (T.L.-N.)

**Keywords:** date fruit, secondary variety, valorization, agricultural by-products, phenolic composition, antioxidant capacity, antimicrobial activity, dietary fiber, prebiotics

## Abstract

Secondary varieties of date fruits are often discarded because they do not have commercial value. However, their phytochemicals are very similar to those of the primary ones and therefore, they can be valorized as a source of compounds of interest, mainly phenols and dietary fiber. Their chemical composition changes with ripening, so their characterization throughout this process is of great significance. Date fruit samples were harvested at Khalal, Rutab, and Tamer stages, and a mixture of fruits from ornamental date trees was also analyzed. Aqueous and ethanolic extracts were studied for their phenolic composition. In aqueous extracts, phenols decreased with ripening, while in the ethanolic ones having higher phenolic content. Chelidonic acid, a γ-pyrone, was the major compound found in all extracts, but in the ethanolic ones, flavonoids were also present in similar amounts. After purification by adsorption chromatography, all extracts were assayed for their antimicrobial activity. Those from the Tamer stage showed the highest activity, especially against Gram-positive bacteria. The fibrous residues after aqueous and ethanolic extractions were also characterized. Their chemical composition suggested that they can be considered as a good source of prebiotic arabinoxylans and antioxidant fiber, whose antiradical activity correlated with their phenolic content. Date fruits from secondary varieties are promising as a worthwhile starting point for obtaining new value-added products.

## 1. Introduction

The consumption of date fruits (*Phoenix dactylifera* L.) is highly recommended for their high nutritional value and health benefits [1]. Dates are particularly rich in polyphenols, which are a type of antioxidant that plays an effective role as an anti-inflammatory factor [1,2] and also displays antibacterial activity [3,4]. These polyphenols improve human health since they reduce the risk of chronic diseases such as heart disease, cancer, and Alzheimer’s disease. In addition to their antioxidant and antimicrobial properties, dates are also an excellent source of dietary fiber, which is essential for maintaining gut health. They can also help to control glycemia and to reduce cholesterol levels in the body [2]. 

While primary date varieties are more commonly known and consumed, secondary date varieties are less popular but equally beneficial. In terms of nutritional content, primary and secondary date varieties are very similar, with each type containing high levels of dietary fiber, antioxidants, and essential minerals such as potassium, magnesium, and iron [5]. However, there are some differences in taste and texture between primary and secondary date varieties [6]. Primary date varieties, such as Medjool and Deglet Noor, have a rich, sweet taste and a soft, chewy texture [7,8]. Both varieties are rich in phytochemicals including antioxidants such as phenolic acids, carotenoids, and flavonoids. These compounds help to protect the body against oxidative stress, which is a major contributor to chronic diseases [1,9,10]. Secondary varieties of dates are equally important sources of phytochemicals, which have been shown to have antioxidant, anti-inflammatory, and anticancer properties.

Understanding the stages of date ripening is crucial for maximizing their nutritional value and phytochemical compositions. The first stage of date ripening is the Kimri stage, which occurs when the date is still green and hard. At this stage, the date is not yet edible and is quite bitter. The second stage is the Khalal stage, which occurs when the date is still crunchy and has a translucent color. At this stage, the date is still not fully ripe but is edible and has a crunchy texture. The third stage of ripening is the Rutab stage, which occurs when the date becomes soft and wrinkly. At this stage, the date is fully ripe and has a soft, chewy texture. The final stage is the Tamer stage, which occurs when the date is fully dried out and has a hard texture. At this stage, the date is still edible and is often used in cooking or as a natural sweetener. Each stage of ripening has its own unique nutritional value and phytochemical composition [11,12]. For example, dates in the Kimri stage are richer in antioxidants than dates in the Tamer stage. When dates are harvested, they are usually unripe and green. At this stage, they are rich in tannins and have a very low sugar content. As the dates ripen, the tannins decrease and the sugar content increases [13,14]. This makes the dates sweeter and more enjoyable to eat. As the dates continue to ripen, their nutritional value increases [11]. Studies have shown that the phytochemical composition of dates changes as they ripen. In unripe dates, the main phytochemicals are phenolic acids and flavonoids [14,15]. As the dates ripen, the concentration of these compounds decreases and the concentration of carotenoids and tocopherols increases [16,17]. Carotenoids and tocopherols are powerful antioxidants, which have been shown to have a wide range of health benefits [14]. In conclusion, the ripening process of dates has a significant impact on their nutritional value and phytochemical composition. It is essential to consider the stage of ripeness when consuming dates for specific nutritional or health benefits. 

The “Palmeral de Elche” is a natural large extension of palm trees within the urban area of the city of Elche (Alicante, Spain) [18]. With more than 70,000 specimens, it is the largest palm grove in Europe, and it is only surpassed by some Arabic oases in the world. In 2000, it was declared a World Heritage Site by UNESCO [19]. There is a native variety from Elche called Confitera, a cultivar with similar characteristics of moisture and texture to Medjool [20]. However, there are also secondary varieties, which are discarded as damaged, blemished, unattractive, unripe, or undersized, which are usually valuable only for cattle feed. Taking into consideration that those secondary dates may be edible and contain many bioactive compounds, misusing them represents an economic loss and generates disposal problems and costs. Few data are available on the compositional and functional characteristics of secondary dates grown in Spain [20]. Efforts are needed to convert these unused Spanish varieties into added-value products with various industrial applications. Therefore, the aim of the present work was to valorize secondary date varieties from Spain at different ripening stages by determining their nutritional and physicochemical characteristics and evaluating their antioxidant and antimicrobial activities. The final aim was to develop new opportunities for using these agricultural co-products as a source of added-value ingredients.

## 2. Results and Discussion

### 2.1. Main Components of Date Pulp 

**Figure 1:**
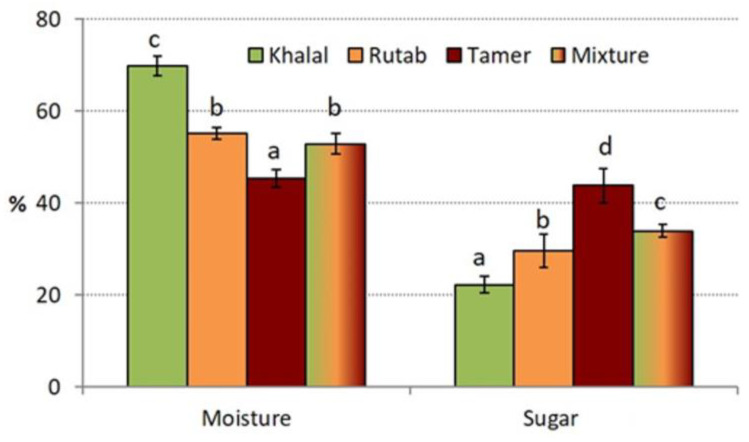
Water and total sugar contents (on fresh weight basis) of the different samples analyzed. The results are the average value of at least three replicates, and the error bars represent standard deviation. Different letters on bars mean significant differences at *p* < 0.05.

The four samples (three maturity stages of a non-commercial variety and a mixture of fruits from ornamental *P. dactylifera* varieties) were analyzed for their moisture and sugar contents, which are the main components of date fruits. As expected from a common ripening process of date fruits, a decrease in moisture and an increase in total sugars were detected between the stages of Khalal and Tamer (Figure 1). The sample identified as Mixture was placed in an intermediate level, very near to the Rutab stage. This similarity can be explained, as they were harvested at a stage between Khalal and Rutab. Moisture decreased from 69.9 to 45.4%. The result for the Tamer stage was near the highest value found in the literature [21] (50.4%), although most date varieties display values between 12 and 35% [22,23]. The moisture levels of Khalal and Rutab were consistent with the results published for other varieties at the same maturity stages [24].

The total sugar contents increased with ripening, from 22% in Khalal to 44% in Tamer (Figure 1). In comparison with three varieties from Iran [24], we found lower values, especially in Rutab and Tamer: in Iranian fruits, the minimum in Rutab was 48% and the maximum in Tamer was 63%, very far from our results. Working with 8 Moroccan varieties [22] and 11 from Tunisia [25], similar results were found for sugar level in Tamer (50–77%). Most of this content was reducing sugars (glucose and fructose, in a ratio near 1), with sucrose being reduced to a very low amount or not present due to the high activity of invertase during fruit ripening [1,22,24,26]. 

For commercial varieties, the ratio between sugar and water contents, known as quality index, is <2 (soft dates), 2–3.5 (semi-soft), and >3.5 (dry dates) [23]. This index is also related to fermentability, and a low score means high fermentation probability and therefore high perishability. That is why date fruits are generally commercialized at an advanced Tamer stage, with low water content, although they could be consumed at earlier maturity stages. The variety studied in this work had a 0.97 quality index at Tamer. This fact, added to its low sugar content, could be the reason for devoting this date fruit production to cattle feed and not to human consumption.

### 2.2. Composition and Antiradical Activity of Date Pulp Extracts

All the samples were extracted with water or 80% ethanol in order to study the composition of soluble bioactive compounds present in the fruit pulp. The results are presented in Table 1.

Comparing the effectiveness of extractants (80% ethanol or water), it is clear that ethanolic extracts were richer than aqueous ones, whatever the maturity stage studied, as described previously [27]. The difference was not only in the total amount of extracted compounds, but also in the kind of compounds identified. Flavonoids accounted for 60–80% of the total phenolic compounds found in ethanolic extracts, but 32–44% in the aqueous ones. In our samples, the total amount of phenols quantified by HPLC-DAD was between 86 and 222 mg/100 g dry weight (DW), 32 and 104 mg/100 g DW of acids, and 32 and 149 mg/100 g DW of flavonoids. Similar amounts were found in dry fruits of the Kharak variety from Iran [28] (141 mg/100 g DW of total phenols and 82 mg/100 g of flavonoids) and in Moroccan Medjool fruits [29] (111 mg/100 g DW of total phenols and 17 mg/100 g of flavonoids). These amounts are significantly higher than others found in the literature [23,30]. It is interesting to note that the sample named Mixture, which included fruits from ornamental *P. dactylifera* varieties, had the highest bioactive compounds content. No general trend was detected in phenolics according to maturity stage.

The total phenolic content in date pulp is usually determined by the Folin—Ciocalteu method in most studies. In this way, there is normally a positive correlation between total phenols and antioxidant activity [31], since the Folin assay could be considered as an antioxidant assay itself. However, when phenolics are quantified by HPLC, this correlation is generally lower or inexistent [23,30,32]. In our case, there were no correlations between total phenols, acids, or flavonoids and antiradical activity, neither in the ethanolic nor in the aqueous extracts. These last findings support the idea that phenolics are not the only compound responsible for antioxidant activity in date fruits. In fact, these fruits are rich in carotenoids, phytosterols, tocopherols, and vitamins (C, B, A, and K) [1,33], which could be also extracted to a different extent by the different used solvents, leading to antioxidant activity levels that do not correlate with phenolic composition.

**Figure 2:**
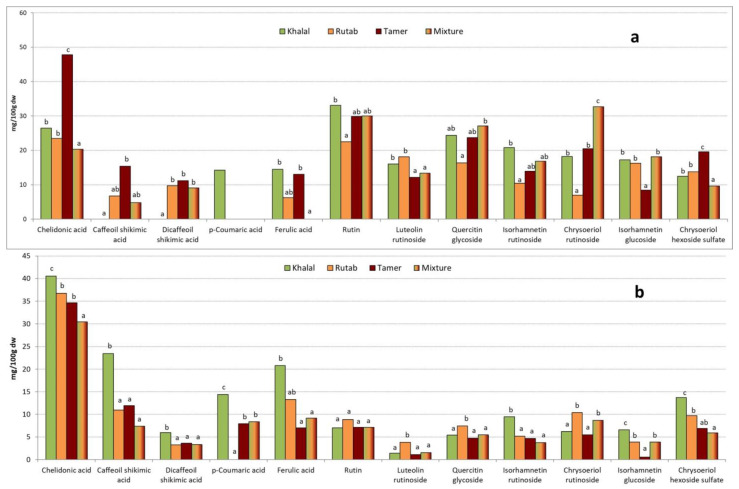
Bioactive compound composition of ethanolic (**a**) and aqueous (**b**) extracts of the four samples analyzed, expressed as mg/100 g dry weight. The same letter means that there are no significant differences (*p* < 0.05) among different maturity stages for a particular phenolic compound.

The compositions of bioactive compounds in both extracts from the four analyzed samples are presented in Figure 2.

Four different phenolic acids, chelidonic acid, and seven flavonoids were identified in all samples. Chelidonic acid was the major compound in all samples and in both extracts. Among flavonoids, rutin was the most abundant in ethanolic extracts, although in the aqueous ones, rutin was in similar percentages to chrysoeriol rutinoside or chrysoeriol hexoside sulfate. Similar results were found when working with Moroccan Medjool date fruits [29] or five Algerian varieties [23]. All the peaks were identified by their UV spectrum and their MS fractionation pattern and compared to the literature. Chelidonic acid, the most abundant aromatic compound in our samples, is a γ-pyrone compound that presents several biological activities such as neurotransmitter regulator, and allergic rhinitis and ulcerative colitis attenuator [34,35,36].

Looking at the composition of ethanolic extracts (Figure 2a), it can be concluded that acids increased with ripening: chelidonic, caffeoyl shikimic, and dicaffeoil shikimic acids increased significantly, coumaric acid was present only in the Khalal stage, and ferulic acid did not show significant differences. The Mixture sample was in almost all cases similar to Khalal–Rutab stages, only the amount of chelidonic acid was significantly lower. In the case of flavonoids, the Khalal stage was the richest, except for chrysoeriol hexoside sulfate, which increased with maturity. The rest of the flavonoids decreased at the Rutab or Tamer stages. The total amount of flavonoids decreased significantly from the Khalal to Rutab stage and then showed an increase at Tamer (150, 108, and 135 mg/100 g DW, respectively). The mixture sample was equivalent to the Khalal stage (148 mg/100 g DW).

### 2.3. Purification of Extracts by Adsorption Chromatography

In order to isolate bioactive compounds and remove sugars from the extracts, they must be fractionated by adsorption chromatography. The behavior of compounds depends on their structural characteristics and interactions with the resin. The resin (Diaion^®^ HP-20) is used in chromatography due to its high porosity, being considered an effective adsorbent for the purification of peptides, proteins, and polyphenols. The recovery of each bioactive compound from ethanolic and aqueous extracts after fractionation is represented in Table 2.

The total recovery of bioactive compounds was between 72% (aqueous extract from Mixture sample) and 88% (ethanolic extract from Tamer maturity stage). Chelidonic acid did not interact with resin and was eluted in a percentage near 100% in the water fraction of all studied samples, together with sugars. This behavior can possibly be attributed to its difference in chemical structure, as chelidonic acid is an aromatic heterocyclic ring—(γ-pyrone)—instead of a phenolic ring.

In almost every sample, the highest recoveries were found in 40% ethanol fractions, where most of the phenolic compounds were eluted. Differences among fractions were higher in the ethanolic extracts, where flavonoids accounted for 60–80% (Table 1). In these extracts, 40% ethanol fractions accounted for 40–70% of the total recovery. It is interesting to highlight the results obtained in the 80% ethanol fraction. In the Khalal sample, no phenolics were quantified, but its percentage increased as the maturity stage progressed (2.37% for Rutab and 7.25% for Tamer). The Mixture sample had the highest percentage (23.87%). These results were not related to the total amount of flavonoids quantified in the initial extracts before purification by chromatography (Table 1), so other factors not yet identified could be affecting the interaction between flavonoids and the resin. This is the reason why all retained fractions (40% ethanol and 80% ethanol fractions) were assayed for their antimicrobial activity, in order to avoid the effects of these uncontrolled factors on subsequent assays.

In general, the recoveries of individual compounds were very high (80–90% and even higher), thus validating the use of Diaion^®^ HP-20 for the purification of phenolic compounds. 

In the case of aqueous extracts, the recoveries of water- and 40% ethanol-eluted fractions were similar due to the lower presence of flavonoids in these extracts than in the ethanolic ones. The 80% ethanol fraction had a very low yield in the four samples analyzed and did not have any relationship with the ripening stages, so it is unlikely it was observed in the ethanolic extracts. However, the recovery of individual compounds was also high, although in almost all cases, these percentages were lower than those from the ethanolic extracts.

In conclusion, the adsorption chromatography step was efficient for the removal of sugars from the extracts, with good recovery for phenolic compounds. Chelidonic acid did not interact with the resin and coeluted with sugars in the water fraction. After this purification, the extracts were ready for the antimicrobial activity assay.

### 2.4. Antimicrobial Activity

#### 2.4.1. Agar Diffusion Test 

The different aqueous and ethanolic date fruit extracts (Khalal, Rutab, Tamer, and Mixture) were tested against Gram-positive and -negative bacteria by the agar diffusion test to determine their potential as antimicrobial agents. The results obtained from this study indicate that all the date fruit extracts (with the exception of the aqueous Mixture date extract) possess antibacterial activity against Gram-positive bacteria (Table 3 and Table 4). 

The ethanolic extracts presented a broad spectrum of activity (Table 3), demonstrating antagonistic activity against a wider range of bacterial species, in comparison with aqueous extracts (Table 4). This result could be attributed to the higher concentration of phenolic compounds found in the ethanolic extracts, as indicated in Table 1, as such compounds are commonly associated with antimicrobial activity [37,38]. Moreover, the Tamer extract was the most promising among the fruit extracts, displaying high antimicrobial activity, which could also be correlated with the higher phenolic content observed in this maturation stage. Amira et al. [11] also have reported that the highest concentration of total phenolic compounds was found in mature firm date fruits, which agrees with the results found herein. Both Gram-positive bacteria belonging to *Bacillus* and *Staphylococcus* genera proved to be sensitive to the Tamer extract (even for a concentration of 100 mg/mL). 

Among the aqueous extracts (Table 4), the Khalal and Rutab extracts only displayed antimicrobial activity in very high concentrations (500 mg/mL). The Tamer date extract was the only one with antimicrobial activity at 100 mg/mL (against bacteria of *Bacillus* genera). In these extracts, a correlation between the phenolic content and a greater antimicrobial activity was not observed.

Each extract was also tested against Gram-negative bacterial species such as *Pseudomonas aeruginosa*, *Klebsiella pneumoniae*, *Agrobacterium tumefaciens*, and *Escherichia coli*. However, no bactericidal activity was observed. In addition, other microorganisms, including *Saccharomyces cerevisiae*, *Alternaria* sp., and *Fusarium oxysporum*, were tested, but none of the extracts displayed antimicrobial activity against these microorganisms.

In general, date palm-based extracts showed antimicrobial activity against both Gram-positive and Gram-negative bacteria [39,40], even though higher sensitivity by Gram-positive bacteria has been reported [41]. Nonetheless, it is worth noting that different factors, such as plant stress conditions and different cultivars [40,42], can influence the production of different metabolites in dates. For this reason, comparison of date extracts from different locations can be challenging, and such variability may account for the detected differences observed in the present study. Additionally, it has been well-documented that Gram-negative bacteria exhibit higher resistance to several antibacterial agents compared to Gram-positive bacteria [43,44,45]. This disparity in susceptibility has been attributed to the presence of an outer lipopolysaccharide membrane. Acting as a permeability barrier, this membrane restricts the uptake of compounds into the bacterial cell. 

The results from the present study agree with previous reports, where it was also noticed that date palm extracts were more efficient against Gram-positive than Gram-negative bacteria [39,46]. This effect was also detected in date palm seed-cake extracts [47]. Furthermore, other studies demonstrated that Gram-positive bacteria are more sensitive to plant extracts than Gram-negative bacteria [48], and this was explained both due to the species-specific structure of the outer membrane and the hydrolytic enzymes produced by these bacteria, which break down the compounds present in the extracts [49,50]. 

#### 2.4.2. Minimum Bactericidal Concentration 

In order to determine the minimum bactericidal concentration (MBC) of each extract, two Gram-positive bacterial species, *S. aureus* and *B. licheniformis*, were selected as representatives. The MBC represents the lowest concentration of an antibacterial agent that significantly reduces the viability of the initial bacterial inoculum. Therefore, a lower MBC value indicates a higher antimicrobial activity of the tested extract. In this study, the results demonstrated that the aqueous extracts generally exhibited higher antimicrobial activity than ethanolic extracts (Table 5).

When comparing both assays (MBC and agar diffusion test) for evaluating antimicrobial activity, some differences were detected. For example, the antimicrobial activities against *S. aureus* were detected in aqueous extracts when using MBC assays, while they were undetectable by agar diffusion tests. Such differences could be due to the diffusion ability of antimicrobial compounds, as the agar diffusion test not only measures the ability of compounds to reduce bacterial growth, but also relies on their ability to diffuse effectively into the agar medium. As a direct contact test, the MBC determination presents advantages over the agar diffusion method [51]. In any case, in both tests, we observed that *B. licheniformis* displayed greater susceptibility to date fruit extracts compared to *S. aureus*, as lower MBC values were found for both aqueous and ethanolic extracts of date fruits. In the case of the aqueous Mixture extract, the MBC value was found to be above 250 mg/mL, which is in accordance with the agar diffusion test, where it was not possible to observe an antimicrobial effect at similar concentrations.

### 2.5. Composition of Fibrous Residues

After aqueous and ethanolic extractions, a fibrous residue remained, which could also be of interest for food applications. Their chemical composition is presented in Table 6.

The yield of fibrous fractions was between 5 and 8% (fresh weight basis). In most cases, there were no significant differences, except for the aqueous extraction of the Tamer stage, which resulted in a significantly lower yield. This fact could be due to the highest content of soluble solids in the most mature fruits, which are more extractable with water rather than with ethanol. This explanation is further supported by the results presented in Table 6, as the fibrous residue obtained from the Tamer stage, whether through aqueous or ethanolic extractions, contained the lowest level of uronic acids, cellulose, and non-cellulosic sugars (NCS). On the other hand, protein, ash, uronic acids, and cellulose were higher in fibers obtained from ethanolic extractions than from aqueous extractions. The content in dietary fiber was remarkably high (71–79% of dry fibrous residue, DFR), which makes these fibrous extracts excellent for the formulation of fiber-enriched foods. These results agreed with the widely accepted consideration of date fruits as a source of dietary fiber [26,33].

Cellulose was the major component of fibrous residues (10.7–24.9% DFR, 18.2% average) except in Tamer and in Mixture aqueous extraction, where it was NCS. However, in general, NCS and uronic acids were present in comparable amounts (13–16.2% DFR, average 14.3% for NCS and 5.5–15.9% DFR, average 12.4% for uronic acids). Protein accounted for 7.3% DFR on average and ashes 2.8%. Lignin, a significant component in date fruit pulp together with cellulose, was not quantified in the present samples. Lignin is always present in these fruits, and its amount can be directly related to fruit edibility [52,53,54]. In this work, lignin presence could be estimated by subtracting cellulose, NCS, and uronic acids from the total dietary fiber value. In this way, lignin varied from 23 to 44% DFR and was always more abundant in the fibrous material after aqueous extraction than following ethanolic extraction, as well as in the most mature samples. The chemical composition of date fruit fiber presented in Table 6 agrees with those presented by other authors [55,56] for date fruits and is aligned with those found in other agricultural by-products considered as good sources of fiber [57,58].

**Figure 3:**
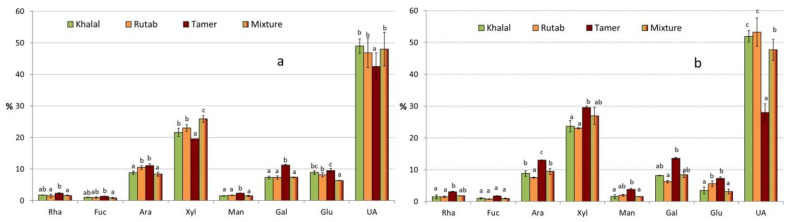
Composition of non-cellulosic sugars of fibrous fractions from ethanolic (**a**) and aqueous (**b**) extracts of the four samples analyzed, expressed in percentages. The same letter means that there are no significant differences (*p* < 0.05) between different maturity stages for a particular sugar and extraction.

The composition of non-cellulosic polysaccharides of four date fruit samples is presented in Figure 3. Pectins could be the major non-cellulosic polymers present in date fruit fiber due to the high percent of uronic acids. Arabinose and galactose could be also linked to this kind of polysaccharide. Among neutral sugars, xylose was the most abundant, pointing to the presence of (arabino)xylans. The quantification of glucose as an NCS near 10% DFR, especially in those fibers from ethanolic extractions, supported the hypothesis raised by some authors about the presence of a β-D-glucan in date fruit pulp with anti-cancer activity [55].

From the presented chemical composition, it could be concluded that date fruit fiber holds potential, not only as a food ingredient in fiber-enriched foods but also as a source of pectins and arabinoxylans [26]. It is widely known that pectins (native, modified, or oligomers) have health effects apart from those linked to dietary fiber, such as anti-inflammatory, for treating cardiovascular diseases and as anti-cancerous agents [59,60]. Nowadays, pectins are being extracted from conventional (citrus and apple) or unconventional sources, mainly agricultural by-products (mango and papaya peels, artichoke, melon peels, olive oil cake, etc.), using environmentally friendly methods (hydrothermal treatments and ultrasound-, microwave-, or enzyme-assisted methods), producing pectic extracts with great health applications [59,61,62,63]. Recently [64], ultrasound extraction was applied to Algerian dates, and the obtained date pectins exhibited in vitro antioxidant and anti-diabetic activities, among other health-promoting capacities. On the other hand, hemicellulose-derived oligosaccharides (HDOs) have proven prebiotic activity with all the benefits that this entails in the alleviation of cardiovascular disease, obesity, type 2 diabetes, and gut diseases (Crohn’s, ulcerative colitis, cancer, diarrhea) [65]. Xylo- and arabinoxylooligosaccharides (XOSs) are examples of HDOs that can be obtained from agricultural by-products since xylans and arabinoxylans are the main hemicelluloses found in lignocellulosic materials [66]; these findings could be considered as a promising starting point for the development of biorefinery processes for the production of XOSs in the framework of the bioeconomy [67]. This concept of second-generation biorefineries, using lignocellulosic biomass as raw material, generates important environmental benefits but does not compete for soil for crop production, which was the main disadvantage of first-generation biorefineries. As far as we know, some approaches have been made to isolate XOSs from date seeds [68] but not from date flesh fiber. A promising research field is open for practical applications.

### 2.6. Phenolics and Antioxidant Activity of Fibrous Residues

Soluble, polymeric, and esterified phenolics have been quantified in the eight fibrous residues obtained (Table 7). In addition, their antiradical capacity was also determined, and correlation studies were done with phenolic content.

The main phenolic group quantified in all samples was the polymeric phenols, which were more abundant in the samples from the aqueous extraction. Only the monomers catechin and epicatechin and their adducts were quantified since a hydrolysis step was necessary to release them. These same components were identified when studying polymeric phenols from date fruit seeds [69,70]. Polymeric phenols, such as the procyanidins, have been studied by other authors [71], and they concluded that polymers up to heptadecamers could be identified by LC-ESI/MS/MS in date fruit pulp from the Deglet Nour variety. As shown in Table 7, procyanidins decreased with maturity in both kinds of fibrous extracts. This fact could be related to the reduction in astringency that occurs as the pulp of date fruit ripens [13,14]. The second group of phenols was the soluble ones. In fibers obtained from aqueous treatments, their content was 3–8 times higher compared to those from ethanolic extraction. This observation correlates well with the lower presence of flavonoids in the aqueous extracts (Table 1). Esterified phenolics were also present as minor compounds, with t-ferulic acid being almost the only p-hydroxybenzoic acid quantified.

The presence of such high amounts of phenols in these fibrous extracts can contribute to their antioxidant activity (Table 7). The antioxidant activity of fibrous extracts from agricultural by-products has been reported in the literature, and the described results are within the range of those presented for Spanish date fruits. For example, working with Tunisian date fruits, Mrabet et al. [52] described a dietary fiber with 38–301 mmol TE/kg antioxidant activity, a capacity that increased up to 550 mmol TE/kg after hydrothermal treatments [53]. Other by-products such as sugar cane molasses [72], okra [73], or citrus fruits [74] are within the same range (around 100, 19–29, and 70–240 mmol TE/kg, respectively) as those presented for Spanish date fruits. The consumption of antioxidant dietary fiber is of great interest, since this fiber carries to the colon a high quantity of antioxidants that exert their protective action in situ on intestinal mucosa, where most gut degenerative processes take place. Table 8 summarizes the results of the regression analysis to assess the relation between antiradical activity and the main groups of phenolics present in date fruit fiber.

All groups, except for esterified phenolics, correlated with antioxidant activity. Since the *p*-values from ANOVA were lower than 0.01, there were significant statistical relationships between free, polymeric, and total phenolics with antioxidant activity with a 99% confidence interval. Unlike ethanolic or aqueous extracts, in the case of date fruit fiber, there was a clear dependence between the different groups of phenolics and antiradical activity.

## 3. Materials and Methods

### 3.1. Plant Material

**Figure 4:**
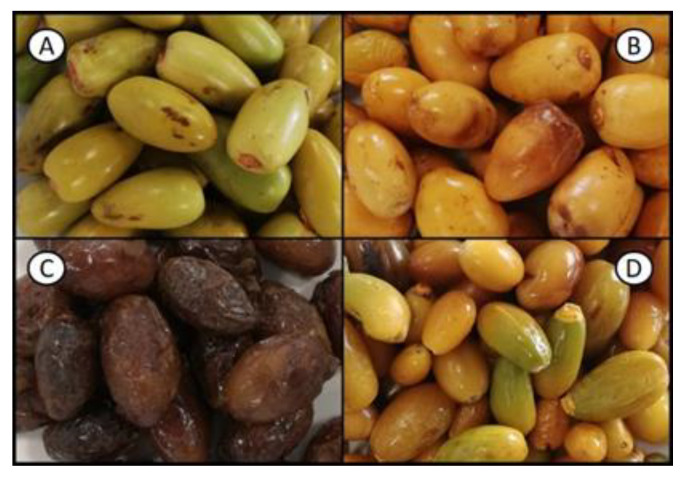
Samples analyzed. (**A**–**C**) are fruits from the same non-commercial *P. dactylifera* variety at different maturation stages ((**A**) Khalal; (**B**) Rutab; (**C**) Tamer) and (**D**) is a mixture of fruits from different ornamental varieties from *P. dactylifera* species harvested at different stages of ripeness.

The four samples of date fruits were purchased from TodoPalmera^®^ (Elche, Spain) [75]. The fruits from one variety of *P. dactylifera* (unknown genetic information), sold as cattle feed, were classified into three ripeness stages (Khalal, Rutab, and Tamer) by TodoPalmera^®^ staff (Figure 4A, B, and C, respectively). Another sample was collected as a blend of different ornamental varieties (all from *P. dactylifera* species), locations, and maturity stages, and was referred to as “Mixture” (Figure 4D). All samples were sent to Instituto de la Grasa (Sevilla, Spain) where they were stored at −20 °C until analysis.

### 3.2. Determination of Moisture and Total Sugars

Ten fruits were pitted, and the flesh was homogenized in a Thermomix^®^ model TM31 (Vorwerk, Spain). One-gram aliquots, in triplicate, were dried in an infrared moisture analyzer (Ohaus, MB45) until constant weight. The results are expressed as %.

The remaining homogenized pulp was extracted twice at a ratio of 1:4 with hot water in the same device, at maximum speed for 1 min. Both extracts were filtered through filter paper and combined. Total sugars were determined from this extract following the anthrone method [76] after preparing the suitable dilution for each sample. The results were expressed as % on dry weight basis.

### 3.3. Obtention and Fractionation of Date Pulp Extracts and Fibrous Residues

Date flesh (150 g in duplicate) was treated twice with 600 mL of extractant (water for aqueous extraction and a mixture 96% ethanol/water for ethanolic extraction). The proportion of water/ethanol varied according to the sample moisture to have a final concentration of 80% ethanol. The homogenates were filtered through cloth filters. The aqueous extracts were directly charged into the chromatographic column, but the ethanolic ones had to be evaporated under vacuum to eliminate ethanol and then filled up to their initial volume with water.

The chromatographic system was composed of a glass column filled with 100 mL (3 cm i.d. × 15 cm height) of Diaion^®^ HP-20, a synthetic adsorbent resin of the highly porous type obtained from Vivaqua International S.L. (Barberà del Vallès, Spain). The resin was activated with 96% ethanol and then equilibrated with distilled water. The extract (1 L) was loaded into the column at a flow rate of 2 bed volumes/h, and the column was sequentially washed with 200 mL water, 400 mL 40% ethanol, and 400 mL 80% ethanol. The initial extracts and the three obtained fractions after adsorption chromatography were analyzed for their phenolic contents by HPLC.

The wet fibrous residues after aqueous/ethanolic extractions were recovered, dried in a recirculating oven at 60 °C for 18 h, weighed, and stored at −20 °C until analysis. 

### 3.4. Analysis of the Phenolic Composition of Pulp Extracts by HPLC

The crude extracts and each chromatographic fraction were analyzed by HPLC [77]. Briefly, the analyses were carried out using a Jasco-LC-Net II ADC liquid chromatograph system (Jasco Inc., Easton, MD, USA) equipped with a diode array detector (DAD). Phenols were separated using a Mediterranea Sea C18 reverse-phase analytical column (25 cm length × 4.6 mm i.d., 5 μm particle size; Teknokroma, Barcelona, Spain). The gradient profile was performed with solvent A (water with 1% formic acid) and solvent B (acetonitrile with 1% formic acid) and developed as follows: from 5% B to 25% B for the first 30 min, to 50% B over the next 15 min, to 100% B over 2 min, to 25% B over the next 3 min, to 5% B over the next 2 min, and finally maintained at 5% B for 3 min (55 min total). The flow rate was 1 mL/min. The spectra from all peaks were recorded in the 200–600 nm range, and the chromatograms were acquired at 360 nm. The quantification of individual phenols was performed using an eight-point regression curve in the range of 0–250 μg on the basis of standards.

### 3.5. Chemical Composition of Date Fibrous Residues

The concentration of protein was determined as % nitrogen content × 6.25 by elemental microanalysis using a Leco CHNS932 analyzer (St. Joseph, MI, USA).

Ashes were determined by incinerating samples in a muffle furnace (Nabertherm model B180, Bremen, Germany) at 550 °C until white ash was obtained.

Uronic acids (UA) were quantified by the m-hydroxybiphenyl method [78] prior to hydrolysis with sulfuric acid [79].

The non-cellulosic sugars (NCS) of each residue were determined by acid hydrolysis with 2 N trifluoroacetic acid (TFA) at 121 °C for 1 h [80], derivatization to alditol acetates, and quantification by gas chromatography [81]. An HP 6890 Plus gas chromatograph (Hewlett-Packard, Palo Alto, CA, USA) fitted with a 30 m × 250 μm × 0.20 mm capillary column (SP-2330, Supelco, Bellefonte, PA, USA) was used. The carrier gas was helium with a constant flow of 2.2 mL/min. Injection was performed in splitless mode. The oven temperature was held at 50 °C for 2 min after injection, then programmed to 180 at 35 °C/min, held at 180 °C for 5 min, then immediately increased to 220 at 5 °C/min, and held at 220 °C for 22 min. Total run was 40.7 min. The injector temperature was 250 °C, and that of the flame ionization detector (FID) was 300 °C.

The cellulose content was determined from the TFA hydrolysis residue by 72% H_2_SO_4_ hydrolysis for 2 h according to Jiménez et al. [82]. The glucose in the hydrolysates was colorimetrically quantified by the anthrone assay [76].

The dietary fiber content was determined using the protocol described by Lee et al. [83] with slight modifications. Briefly, 1 g replicates (in duplicate) of dry fibrous residue were suspended in 40 mL of MES (2-(N-morpholino)ethanesulfonic acid)-Tris buffer and treated with 50 mL of Thermamyl (heat-stable a-amylase) at 100 °C for 15 min and then digested with 100 mL of a 50 mg/mL protease solution (60 °C, 30 min), followed by incubation with 100 mL of amyloglucosidase (60 °C, 1 h) to remove protein and starch. Then, four volumes of 96% hot ethanol were added to the hydrolysate, and the total volume was passed through the sintered glass crucible (no. 2) using the Fibertec E system (1023 filtration module). The retained fiber was dried overnight at 105 °C in an air oven and weighed. Protein and ash were determined from fiber residue for weight correction.

### 3.6. Phenolic Composition of Fibrous Residue

The free phenolics were extracted from the fibrous residues with two treatments of 80% ethanol at a ratio of 1:4 *w*/*v*. The phenolic composition was determined by HPLC according to the method previously described for phenolic compound determination from pulp extraction.

For quantifying polymeric phenolics, the samples were subjected prior to phloroglucinolysis [68]. Each fibrous sample (50 mg ×2) was mixed with 800 μL of phloroglucinolysis solution (0.1 N methanolic HCl, containing 50 mg/mL phloroglucinol and 10 mg/mL ascorbic acid). The mixture was vortexed and incubated at 50 °C for 20 min. Then, the samples were cooled in ice, and 1 mL of 40 mM sodium acetate was added. The samples were centrifuged at 217× *g* for 5 min, and the supernatant was analyzed by HPLC. The equipment and column were the same as previously described. The gradient profile was performed with solvent A (water with 1% formic acid) and solvent B (acetonitrile) and developed as follows: from 0% B to 100% B for 45 min, maintained at 100% B over the next 5 min, back to 0% B over 5 min, and maintained at 0% B for 5 min (60 min total). The flow rate was 1 mL/min. Spectra from all peaks were recorded in the 200–600 nm range, and the chromatograms were acquired at 360 nm. The quantification of individual phenols was performed using an eight-point regression curve in the range of 0–250 μg on the basis of standards.

Esterified phenols were quantified by HPLC after saponification [84]. Samples (in duplicate) were treated with 2 M NaOH for 24 h, at room temperature, under nitrogen in the dark. After filtration, t-cinnamic acid was added as the internal standard; the solutions were acidified to pH < 2 with concentrated HCl and then extracted three times with ethyl acetate (three volumes). The ethyl acetate extracts were evaporated to dryness under nitrogen and then dissolved in 200 μL of 50% (*v*/*v*) aqueous methanol prior to analysis by HPLC. The individual compounds were detected and quantified by HPLC using the same equipment and column as previously described. The gradient profile for the separation of esterified phenolics was formed using solvent A (10% aqueous acetonitrile, 2 mL/L acetic acid) and solvent B (40% methanol, 40% acetonitrile, 20% water, 2 mL/L acetic acid) in the following program: initially A 90%, B 10%; linear gradient over 17 min to A 57.5%, B 42.5%; held at A 57.5%, B 42.5% for a further 6 min; linear gradient over 17 min to B 100%; linear gradient over 5 min to A 90%, B 10%; held at A 90%, B 10% for a further 5 min. The flow rate was maintained at 1 mL/min. Quantification of compounds was performed by integration of peak areas at 280 nm with reference to calibrations made with known amounts of pure compounds.

### 3.7. Antioxidant Activity

The antiradical activity of the different ethanolic and aqueous extracts was determined by the DPPH· method [85]. The efficient concentration EC_50_ was calculated from a calibration curve by linear regression for each sample.

The antiradical activity of the fibrous residue after ethanolic/aqueous extraction was evaluated by the QUENCHER-DPPH· method, as described by Serpen et al. [86], with some modifications [87]. As for the extract antiradical activity, EC_50_ was also calculated. Both activities were expressed as millimoles of Trolox equivalent antioxidant capacity per kilogram of sample by means of a dose–response curve for Trolox.

### 3.8. Antimicrobial Assay of Phenolic Extracts

#### 3.8.1. Microorganisms and Growth Conditions

All the microorganisms used in this study were obtained from the Center of Molecular and Environmental Biology collection (University of Minho) and included *S. aureus* ATCC 6538, *S. epidermidis* ATCC 35984, *A. tumefaciens* GB3101, *E. coli* DH10β, *P. aeruginosa* ATCC 10145, *K. pneumoniae* 2978, *B. subtilis* UH1093, *B. licheniformis* UH109, and *B. rugosus* UH193. All bacterial strains were cultivated in Luria Bertani (LB) media or LB agar plates obtained by adding 15 g/L of agar to LB. *S. cerevisiae* AH109 was used as a yeast representative and was grown in Yeast-Peptone-Dextrose media (YPD) or YPD agar (20 g/L). *F. oxysporum* cab77 and *Alternaria* sp. Mn11 were cultivated in Potato Dextrose Agar (PDA, Liofilchem).

#### 3.8.2. Preparation of Date-Based Extracts 

After adsorption chromatography purification, the eight phenolic extracts (aqueous and ethanolic extracts from Khalal, Rutab, Tamer, and Mixture) were concentrated under vacuum and freeze dried. The solution of each extract was prepared by dissolving each extract powder in 10% (*v*/*v*) dimethyl sulfoxide (DMSO) at concentrations ranging from 25–500 mg/mL under sterile conditions and incubating at 60 °C for 2 h.

#### 3.8.3. Evaluation of Antimicrobial Activity by Diffusion Test Assay

To assess the antimicrobial activity of the extracts, the agar diffusion test was conducted [88]. All bacterial/yeast strains were cultured overnight at a temperature of 30 °C and an agitation of 200 rpm (Stuart S150 orbital incubator). The optical density (OD) at 600 nm was measured using a spectrophotometer (Multiskan Plus MKII, Titertek) and adjusted to 0.2–0.3 with culture media (approximately 108 CFU/mL). Then, 100 μL of each suspension was spread onto LB plates or YPD agar. Filter paper discs (area of 19.63 mm^2^) were distributed in different regions of the plate, and 15 μL of each extract with concentrations ranging from 25 to 500 mg/mL were added on top of each paper disc. The plates were incubated overnight at 30 °C. The area of the inhibition zone was evaluated using the Image J 1.53k software (USA). 

For determining the antimicrobial activity against fungal species, the fungi were first cultured at a temperature of 30 °C for 6 days or until spore formation occurred. The spores were isolated by collecting the mycelium from the plates in 10 mL of ultrapure water and filtered with CalbiochemR Miracloth (EMD Millipore, Burlington, MA, USA). Then, sequential centrifugations were performed at 3500× *g* for 5 min to wash spores and adjust the final concentration to 1 × 10^6^ spores/mL. This was accomplished by using a Neubauer chamber for accurate measurements. Each spore suspension (200 μL) was spread onto PDA plates, and the evaluation of the antimicrobial activity of studied extracts was performed as described above for bacteria and yeast. The plates were incubated at 30 °C and observed daily up to the sixth day. 

#### 3.8.4. Minimum Bactericidal Concentration

The minimum bactericidal concentration (MBC) is defined as the lowest concentration of an antibacterial agent that reduces the viability of the initial bacterial inoculum by ≥99.9% [89]. MBC was determined by the broth microdilution method, using *S. aureus* and *B. licheniformis* as representatives. For this, bacteria were cultured in Mueller Hinton (MH) medium overnight at a temperature of 30 °C and an agitation of 200 rpm (Stuart S150 orbital incubator). The OD of each overnight culture was adjusted to 3–7 × 10^8^ CFU/mL (OD ≅ 0.2–0.4). The different studied date extracts were 2-fold serially diluted in MH, resulting in concentrations ranging from 1 to 250 mg/mL. Dilutions were distributed (100 μL) into the wells of a 96-well plate, and 10 μL of each bacterial suspension was added to each well to obtain a final concentration of 3–8 × 10^5^ CFU/mL. Wells with DMSO plus bacteria in MH, bacteria in MH, and only extracts were used as controls. The 96-well plates were incubated for 24 h at 30 °C, and then each bacterial suspension (100 μL) was plated on MH agar plates and incubated again for 24 h under the same conditions.

### 3.9. Statistical Analysis

The results are expressed as the average value of at least three repetitions ± standard deviation. To assess the differences among samples, a multiple-sample comparison was performed using the Statgraphics Centurion 19 program. Multivariate analysis of variance (ANOVA), followed by Duncan’s multiple comparison test, were performed to differentiate among the groups (*p* < 0.05). Correlation coefficients (R) were determined using regression analysis at the same confidence level.

## 4. Conclusions

According to the circular bioeconomy, there are no agricultural residues, but there are chances for new production lines. In line with this view, secondary date varieties bring the possibility of obtaining value-added extracts. Phenolic extracts could be isolated by aqueous or ethanolic treatments. Looking at their composition, the ethanolic ones contained higher amounts of phenolic compounds than the aqueous ones, especially flavonoids. Their antioxidant activity was also higher in the ethanolic extracts. However, due to the complex composition of bioactive compounds in date palm, no correlation was found between phenolics and antioxidant activity, indicating the need for deeper investigations on extracts characterization. The ripening process did not impact the phenolic composition or antioxidant activity of dates. It is interesting to point out that the sample composed of a mixture of date fruits from ornamental varieties showed the highest scores for both parameters, broadening in this way the potential application fields for date fruit valorization. Whether obtained through ethanolic or aqueous extractions, phytochemicals can be easily purified by adsorption chromatography. This process results in a non-retained fraction composed mainly of sugars, where only chelidonic acid was detected. In the ethanol-eluted fractions, a high percentage of phenolics was recovered. These purified ethanolic extracts have been shown to exhibit antimicrobial activity, especially against Gram-positive bacteria. This activity increased as date fruit ripened, with the ethanolic extracts showing a broader spectrum than the aqueous ones.

The use of liquid extracts is not the only way for giving an added value to date fruit from secondary varieties. The remaining fibrous solids obtained after water/ethanol extraction have also revealed interesting characteristics. Their chemical composition makes them good candidates as ingredients for the formulation of fiber-enriched foods. In addition, and considering their individual components, they can be explored as feedstock for obtaining bioactive oligosaccharides from pectins and/or arabinoxylans. Their antioxidant activity, which correlates with the phenolic content, is another attribute that can be also explored since the consumption of this antioxidant fiber could contribute to gut health.

With this research, we are opening a wide field of work for researchers and industries because the findings and conclusions of this study can be applied, not only to Spanish date fruit, but also to worldwide production of all edible, secondary, and even ornamental date palm varieties. Biorefineries could be developed to value sugars, phenols, and fiber from date by-products, which now have very limited use, but could become worthy materials for various production areas.

## Figures and Tables

**Table 1:** Composition of soluble bioactive compounds and antiradical activity in ethanolic and water extracts from date fruit pulp. Total amounts are expressed as mg/100 g dry weight and the antiradical activity as mmol Trolox equivalent/kg dry weight).

	Khalal	Rutab	Tamer	Mixture
**Ethanolic extract**				
Total phenolics	205.12 ± 1.39 c B	154.42 ± 3.01 a B	222.42 ± 3.36 d B	182.41 ± 8.71 b B
% acids	26.97 ± 1.65 b	29.96 ± 2.05 b	39.34 ± 1.13 c	18.66 ± 5.01 a
% flavonoids	73.03 ± 1.65 b	70.04 ± 2.05 b	60.66 ± 1.13 a	81.34 ± 5.01 c
Antiradical activity	6.24 ± 0.06 b A	16.02 ± 0.42 c B	1.29 ± 0.04 a B	59.28 ± 3.41 d B
**Aqueous extract**				
Total phenolics	162.80 ± 10.10 c A	114.67 ± 3.88 b A	96.23 ± 7.57 ab A	85.90 ± 5.00 a A
% acids	64.63 ± 0.75 bc	55.95 ± 3.34 a	67.69 ± 1.08 c	58.86 ± 3.18 ab
% flavonoids	35.37 ± 0.75 ab	44.05 ± 3.34 c	32.31 ± 1.08 a	41.14 ± 3.18 bc
Antiradical activity	6.21 ± 0.13 c A	4.13 ± 0.14 b A	0 a A	10.53 ± 0.49 d A

The same lower-case letter means that there are no significant differences (*p* < 0.05) among different maturity stages in the same kind of extract. The same capital letter means that there are no significant differences (*p* < 0.05) between different extracts from the same sample.

**Table 2:** Recoveries (%) and distribution of the different bioactive compounds after adsorption chromatography. “Total” expresses the percentage that the fraction represents in relation to the total amount quantified before purification.

Ethanolic Extracts	Khalal			Rutab			Tamer			Mixture		
	Water	40%EtOH	80%EtOH	Water	40%EtOH	80%EtOH	Water	40%EtOH	80%EtOH	Water	40%EtOH	80%EtOH
Chelidonic acid	95.74			101.09			98.48			78.30	11.28	
Caffeoil shikimic acid		74.52			44.18			99.91		24.01		
Dicaffeoil shik. acid		78.02			40.82			63.54	32.99		75.92	
Coumaric acid		81.71										
Ferulic acid		79.75			63.75			70.34			70.10	
Rutin		79.65			70.57	2.94		85.44			48.08	25.57
Luteolin rutinoside		86.50			70.18	0.86		86.42	7.58			
Quercitin glycoside		86.53			74.30	5.59		62.53	12.68		35.82	53.88
Isorhamnetin rutinoside		81.70			80.50	1.34		71.30	11.28		41.25	35.15
Chrysoeriol rutinoside		70.62			83.79	6.47		88.39	5.52		43.76	20.27
Isorham. glucoside		71.62			75.52			92.984			41.82	44.39
Chrys. hexoside sulfate		85.98			88.00	6.51		87.05	8.03		44.95	27.03
**Total**	**11.99**	**70.20**	**-**	**14.34**	**61.18**	**2.37**	**9.15**	**72.73**	**7.25**	**15.57**	**40.37**	**23.87**
**Aqueous Extracts**	**Khalal**			**Rutab**			**Tamer**			**Mixture**		
	Water	40%EtOH	80%EtOH	Water	40%EtOH	80%EtOH	Water	40%EtOH	80%EtOH	Water	40%EtOH	80%EtOH
Chelidonic acid	101.87	5.58		95.39	1.41		107.88			87.97		
Caffeoil shikimic acid		46.78			1.43		12.88	43.80				
Dicaffeoil shik. acid		35.18			4.88							
Coumaric acid	63.65	33.93					51.27	33.86			45.12	
Ferulic acid	63.45	35.48		41.22	5.35			42.22			41.43	
Rutin		101.18			84.79			62.00			95.66	10.88
Luteolin rutinoside												
Quercitin glycoside		92.65			101.03			64.12			68.05	16.75
Isorhamnetin rutinoside		58.64	20.27					64.61			82.97	21.92
Chrysoeriol rutinoside		79.43	16.55		66.63						60.16	17.55
Isorham. glucoside		73.18	27.12		90.79						46.08	40.68
Chrys. hexoside sulfate		96.68	3.16		92.47						64.79	44.06
**Total**	**40.76**	**43.6**	**3.75**	**35.39**	**39.10**	**-**	**50.24**	**30.15**	**-**	**28.63**	**34.34**	**8.80**

**Table 3:** Area of inhibition zones (cm^2^) ± SD obtained in the agar diffusion test when the ethanolic extracts (concentration in mg/mL) were tested against several Gram-positive (*Bacillus subtilis*, *B. licheniformis*, *B. rugosus*, *Staphylococcus aureus*, and *S. epidermidis*) bacteria.

	Concentration	*B. licheniformis*	*B. subtilis*	*B. rugosus*	*S. aureus*	*S. epidermidis*
Khalal	500	0.814 ± 0.167	0.568 ± 0.116	0.549 ± 0.082	1.008 ± 0.037	0.727 ± 0.064
	250	0.572 ± 0.055	0.472 ± 0.152	Nt	Na	Nt
Rutab	167	0.701 ± 0.076	0.534 ± 0.030	Na	0.792 ± 0.068	Nt
	150	Na	Na	Na	Na	Nt
Tamer	250	0.755 ± 0.080	0.702 ± 0.090	Nt	0.707 ± 0.090	Nt
	100	0.515 ± 0.025	0.703 ± 0.090	Nt	0.531 ± 0.021	Nt
Mixture	500	0.700 ± 0.102	0.684 ± 0.106	0.561 ± 0.041	0.985 ± 0.103	0.998 ± 0.185
	250	Na	Na	Na	Na	Na

Na: no activity; Nt: not tested.

**Table 4:** Inhibition zone area (cm^2^) ± SD in the agar diffusion test of aqueous extracts (concentration in mg/mL) against the same bacterial species tested for ethanolic extracts (Table 3).

	Concentration	*B. licheniformis*	*B. subtilis*	*B. rugosus*	*S. aureus*	*S. epidermidis*
Khalal	500	0.727 ± 0.064	0.554 ± 0.195	0.336 ± 0.045	Na	Nt
	250	Na	Na	Na	Na	Nt
Rutab	500	1.104 ± 0.030	0.815 ± 0.144	0.597 ± 0.055	Na	Nt
	250	Na	Na	Na	Na	Nt
Tamer	250	0.724 ± 0.081	0.800 ± 0.153	0.706 ± 0.097	Na	Na
	100	0.642 ± 0.020	0.417 ± 0.024	0.475 ± 0.065	Na	Na
Mixture	250	Na	Na	Na	Na	Nt

Na: no activity; Nt: not tested.

**Table 5:** Minimum bactericidal concentration (in mg/mL) of different date extracts when tested against two Gram-positive bacterial species.

	Solvent	*B. licheniformis*	*S. aureus*
Khalal	Aqueous	62.5	62.5
	Ethanolic	62.5	125
Rutab	Aqueous	31.3	62.5
	Ethanolic	125	125
Tamer	Aqueous	31.3	125
	Ethanolic	62.5	62.5
Mixture	Aqueous	125	>250
	Ethanolic	>250	>250

**Table 6:** Chemical composition of fibrous residues obtained after aqueous and ethanolic extractions. The yield is expressed as g/100 g date fruit fresh weight and the chemical component as g/100 g of dry fibrous residue.

	Khalal	Rutab	Tamer	Mixture
Fiber from ethanolic extraction				
Yield	7.68 ± 0.24 a A	7.70 ± 0.01 a A	6.60 ± 8.01 a B	8.01 ± 0.72 a A
Protein	7.17 ± 0.04 a B	7.37 ± 0.01 a B	11.20 ± 0.14 c B	7.80 ± 0.21 b B
Ash	3.73 ± 0.29 a B	3.62 ± 0.12 a B	5.29 ± 0.21 b B	3.92 ± 0.06 a B
Uronic acids	13.12 ± 0.61 b A	12.50 ± 1.23 b A	9.59 ± 0.97 a B	14.92 ± 1.65 c A
Cellulose	23.33 ± 1.78 bc A	24.91 ± 2.13 c B	16.83 ± 1.30 a B	20.87 ± 1.50 b B
Non-cellulosic sugars	13.68 ± 0.60 ab A	14.20 ± 1.20 ab A	12.97 ± 0.62 a A	16.17 ± 1.49 b A
Dietary fiber	74.25 ± 1.03 b A	74.94 ± 0.30 b A	71.49 ± 1.72 a A	75.81 ± 0.59 b A
**Fiber from aqueous extraction**				
Yield	7.73 ± 0.19 b A	7.65 ± 0.06 b A	5.15 ± 0.29 a A	8.30 ± 0.47 b A
Protein	5.99 ± 0.00 b A	5.77 ± 0.04 a A	7.03 ± 0.01 d A	6.26 ± 0.03 c A
Ash	1.99 ± 0.04 b A	0.93 ± 0.30 a A	1.66 ± 0.02 b A	1.64 ± 0.18 b A
Uronic acids	15.94 ± 0.56 c B	14.23 ± 1.18 b A	5.56 ± 0.55 a A	13.31 ± 0.93 b A
Cellulose	21.25 ± 2.92 c A	15.43 ± 1.11 b A	12.31 ± 0.95 a A	10.67 ± 0.74 a A
Non-cellulosic sugars	14.76 ± 0.12 b A	13.39 ± 1.25 a A	14.32 ± 0.70 a A	14.59 ± 0.03 b A
Dietary fiber	79.25 ± 1.23 a B	77.43 ± 1.16 a A	76.84 ± 0.71 a A	79.17 ± 1.69 a A

The same lowercase letter means that there are no significant differences (*p* < 0.05) between different maturity stages in the same kind of extract. The same capital letter means that there are no significant differences (*p* < 0.05) between different extract from the same sample.

**Table 7:** Phenolic composition of fibrous residues obtained after aqueous and ethanolic extractions from the four samples studied. The amount of phenolics is expressed as mg/100 g dry fibrous residue and the antiradical activity as mmol Trolox Equivalent/kg dry fibrous residue.

	Khalal	Rutab	Tamer	Mixture
Fiber from ethanolic extraction				
Soluble phenolics	87.79 ± 2.67 a A	103.89 ± 3.72 b A	85.52 ± 0.32 a A	109.89 ± 8.89 b A
Phenolic acids (%)	20.07 ± 2.21 a	19.10 ± 0.54 a	33.10 ± 0.77 b	21.04 ± 0.89 a
Flavonoids (%)	79.92 ± 2.21 b	80.90 ± 0.54 b	66.90 ± 0.77 a	78.96 ± 0.89 b
Polymeric phenolics	3040.74 ± 32.71 d A	2476.11 ± 29.55 c A	1019.36 ± 5.03 a A	2124.32 ± 2.51 b A
Epicatechin adduct (%)	73.45 ± 0.00 d	66.53 ± 1.05 c	31.13 ± 0.70 a	63.96 ± 0.70 b
Catechin (%)	20.18 ± 0.32 a	24.47 ± 0.92 b	51.54 ± 0.48 d	27.96 ± 0.36 c
Epicatechin (%)	6.37 ± 0.32 a	9.00 ± 0.14 c	17.33 ± 0.22 d	8.08 ± 0.34 b
Esterified phenolics	39.52 ± 0.67 a A	65.51 ± 3.50 d A	56.21 ± 2.09 c B	49.85 ± 3.48 b A
p-Coumaric acid (%)		17.52 ± 0.63		
t-Ferulic acid (%)	100	82.48 ± 0.62	100	100
Total phenolics	3168.05 ± 36.05 d A	2645.51 ± 36.77 c A	1161.09 ± 7.44 a A	2284.06 ± 14.88 b A
Antiradical activity (mmolTE/Kg DW)	32.77 ± 4.03 b	35.67 ± 3.70 b	21.81 ± 0.68 a	46.22 ± 5.54 c
**Fiber from aqueous extraction**				
Soluble phenolics	477.26 ± 18.85 b B	343.87 ± 15.60 a B	699.78 ± 30.08 d B	597.90 ± 23.97 c B
Phenolic acids (%)	1.47 ± 0.05 a	4.84 ± 0.62 c	6.82 ± 0.07 d	3.86 ± 0.19 b
Flavonoids (%)	98.51 ± 0.05 d	95.15 ± 0.62 b	93.17 ± 0.07 a	96.14 ± 0.19 b
Polymeric phenolics	3451.59 ± 228.26 b A	3475.28 ± 171. 44 b B	3067.11 ± 27.05 ab B	2717.87 ± 74.54 a B
Epicatechin adduct (%)	83.64 ± 0.46 b	82.21 ± 0.14 b	79.74 ± 1.41 a	82.05 ± 0.59 b
Catechin (%)	10.25 ± 0.29 a	10.83 ± 0.39 a	12.20 ± 0.69 b	9.82 ± 0.44 a
Epicatechin (%)	6.16 ± 0.75 a	6.96 ± 0.54 ab	8.06 ± 0.72 b	8.13 ± 0.15 b
Esterified phenolics	42.64 ± 0.05 a B	46.53 ± 1.31 b A	50.23 ± 1.77 c A	46.52 ± 1.79 b A
t-Ferulic acid (%)	100	100	100	100
Total phenolics	3971.59 ± 247.16 a B	3865.68 ± 188.35 a B	3817.12 ± 58.9 a B	3362.29 ± 100.30 a B
Antiradical activity (mmolTE/Kg DW)	142.57 ± 7.94 c	113.90 ± 14.09 b	62.15 ± 2.25 a	75.52 ± 7.71 a

The same lowercase letter means that there are no significant differences (*p* < 0.05) between different maturity stages in the same kind of extract. The same capital letter means that there are no significant differences (*p* < 0.05) between different extracts from the same sample.

**Table 8:** Regression analysis between antioxidant activity and the different groups of phenolics.

	Model	r^2^	*p*-Value
Free phenolics	y=1/(a+b/x)	76.34%	0.0046
Polymerics	y=exp (a+b/x)	71.30%	0.0084
Esterified	$y=a+b\sqrt{x}$	23.58%	0.2225
Total	y=1/(a+bx)	78.77%	0.0033

## Data Availability

The data presented in this study are available on request from the corresponding author.
